# “When Somebody Comes into This Country and You Are Trans on Top of That Is Like You Got… Two Strikes on You”: Intersectional Barriers to PrEP Use Among Latina Transgender Women in the Eastern and Southern United States

**DOI:** 10.3390/ijerph22050659

**Published:** 2025-04-22

**Authors:** Rodrigo A. Aguayo-Romero, Genesis Valera, Erin E. Cooney, Andrea L. Wirtz, Sari L. Reisner

**Affiliations:** 1 The Institute for Health Research & Policy at Whitman Walker, Washington, DC 20009, USA; 2 The Fenway Institute, Fenway Health, Boston, MA 02215, USA; gvalera@umich.edu; 3 Department of Epidemiology, University of Michigan School of Public Health, Ann Arbor, MI 48109, USA; sreisner@umich.edu; 4 Department of International Health, Johns Hopkins Bloomberg School of Public Health, Baltimore, MD 21205, USA; ecooney2@jhmi.edu (E.E.C.); awirtz1@jhu.edu (A.L.W.); 5 Center for Public Health and Human Rights, Department of Epidemiology, Johns Hopkins Bloomberg School of Public Health, Baltimore, MD 21205, USA

**Keywords:** HIV, HIV prevention, transgender, pre-exposure prophylaxis, intersectionality, stigma, discrimination

## Abstract

In the United States (U.S.), Latina transgender women (LTW) are highly burdened by HIV and are prioritized for pre-exposure prophylaxis (PrEP). This study explored intersectional barriers and facilitators to PrEP uptake among LTW. Between February–November 2022, in-depth interviews were conducted with 27 LTW in the LITE Study. Participants were purposively sampled from 196 LTW in the cohort based on PrEP uptake (PrEP-naïve n = 8, PrEP-eligible and not user n = 5, current PrEP user n = 6, previous PrEP user n = 8). We conducted content analysis guided by a Modified Social Ecological Model and Intersectionality Framework. The mean age of participants was 32.3 (SD = 12.9). Themes were: (1) Intrapersonal: Medical distrust, acceptability of PrEP modalities, and concerns about long-term health; (2) Interpersonal: Mistreatment in healthcare, discrimination-related healthcare avoidance, difficulty finding trans-competent providers, language barriers, and shame and stigma; and (3) Structural: PrEP in the context of limited access to gender-affirming care and widespread silicone use, immigration status, economic marginalization, lack of community outreach, transphobia and anti-transgender legislative contexts, and xenophobia. This study found multilevel intersectional barriers influence PrEP uptake and persistence. Culturally tailored HIV prevention efforts are needed to address LTW-specific barriers, provide information on programs subsidizing PrEP, and implement policy change to ensure equitable PrEP access.

## 1. Introduction

In the United States (U.S.), transgender women are highly burdened by HIV [1,2] and are a priority population in the national strategy to end the HIV epidemic [3], which aims to reduce new HIV infections by 90% by 2030 [4]. The U.S. Centers for Disease Control and Prevention (CDC)’s most recent 2019–2020 National HIV Behavioral Surveillance (NHBS) report documented that more than four out of every 10 (42.2%) transgender women in seven major cities were living with HIV [5]. HIV incidence was most recently estimated at 5.5/1000 person-years (95%CI: 2.7–8.3) in a cohort of transgender women living in the Eastern and Southern US, with increased rates among racial and ethnic minority groups [6]. In the 2019-20 NHBS, Latina transgender women made up 35.0% of newly diagnosed HIV cases among transgender women [7]. This alarmingly high HIV prevalence and incidence highlights the need for evidence-based and tailored HIV prevention strategies for Latina transgender women, including those which integrate biomedical prevention methods such as pre-exposure prophylaxis (PrEP).

Although PrEP agents, including a daily oral pill and long-acting injectable (LAI), have been shown to be efficacious in preventing HIV acquisition among transgender women [8,9], PrEP uptake remains low [7,10]. Among HIV-negative Latina transgender women surveyed across seven U.S. cities in the 2019–2020 National HIV Behavioral Surveillance (NHBS), 90.4% had heard of PrEP, 36.9% reported PrEP use in the last 12 months, and 16.7% reported condomless sex with an HIV-discordant partner at last sex encounter [7]. Despite a high need for PrEP, there continues to be a gap between PrEP indications and uptake for Latina transgender women [11,12,13]. Further, challenges with PrEP adherence and high PrEP discontinuation rates have been found among Latina transgender women [12,14,15]. Medication adherence is especially important to the effectiveness of daily oral PrEP for transgender women [16]. Therefore, while PrEP is available to Latina transgender women, there is a need to understand the context surrounding PrEP uptake and adherence for Latina transgender women, including exploring the perceived acceptability of new and emerging PrEP modalities among this population.

The Modified Social Ecological Model has been used to assess the multiple ecological contexts in which HIV vulnerabilities occur for populations who are disproportionately burdened by HIV [17], including transgender people [18]. It has also been used to conceptualize the role of stigma in health inequities for transgender subgroups such as minoritized racial and ethnic transgender people [19]. Transgender women of color experience multilevel drivers of vulnerability for HIV acquisition, including at intrapersonal (e.g., medical mistrust), interpersonal (e.g., stigma in healthcare), and structural (e.g., employment discrimination) levels [18]. Consistent with the Intersectionality Framework [20,21], Latina transgender women experience multiple forms of oppression that contribute to HIV morbidity, driven by interlocking systems of power that systematically privilege and devalue their intersectional social positions (e.g., racism, transphobia, xenophobia). The multilevel, intersectional factors that fuel the HIV epidemic for Latina transgender women and result in high HIV incidence rates also adversely impact HIV prevention services and PrEP uptake among Latina transgender women [18].

Prior research has found multiple barriers to HIV prevention services and PrEP uptake for transgender women, including for Latina transgender women [22,23]. At the intrapersonal level, transgender women experience general medical mistrust [22,24,25,26,27] and distrust related to PrEP specifically [11]. At the interpersonal level, stigma in healthcare, such as experiencing mistreatment, misgendering, and difficulties finding a trans-friendly provider, can lead to avoidance of healthcare settings and services for Latina transgender women [12,28]. Stigma and shame related to sexual behaviors, or stereotypes associated with being “at risk”, also deter Latina transgender women from seeking services [13]. At the structural level, documented barriers to PrEP include economic marginalization, immigration status, lack of access to gender-affirming healthcare systems, and lack of culturally responsive outreach to Latina transgender women [11,12,29]. However, additional research is needed to further elucidate barriers to uptake, adherence, and persistence, including perceptions of different and emerging PrEP modalities, and to identify strategies for optimal outreach, information, and PrEP navigation to meet the unique needs of Latina transgender women. Further, previous studies have primarily sampled Latina transgender women from a specific geographic locale (e.g., Los Angeles, Baltimore–Washington, DC) [11,29], rather than across multiple geographic contexts to assess shared experiences.

The alarmingly high prevalence and incidence of HIV among Latina transgender women [1,2] underscore the urgent need for tailored and evidence-based HIV prevention strategies that incorporate biomedical methods like pre-exposure prophylaxis (PrEP). Despite the availability of PrEP, uptake and adherence remain notably low within this community [11,12,13,14,15], necessitating a deeper understanding of the contextual factors involved. The multifaceted and intersectional barriers contributing to the HIV epidemic among Latina transgender women also hinder access to HIV prevention services and PrEP usage. To address this, additional research is needed to explore these barriers, and to develop effective outreach and navigation strategies tailored to their unique needs. It is critical to conduct studies that encompass diverse geographic locales to better capture the shared experiences and challenges faced by Latina transgender women across different regions. Applying a Modified Social Ecological Model [17,18] and Intersectionality Framework [20,21,30], this qualitative study sought to explore the multilevel and intersectional barriers and facilitators to PrEP uptake and persistence among a sample of Latina transgender women in the Eastern and Southern U.S.

## 2. Materials and Methods

### 2.1. Participants and Procedures

The Leading Innovation for Transgender Women’s Health and Empowerment (LITE) Study was a multisite hybrid cohort of racially and ethnically diverse transgender women across the Eastern and Southern U.S. who were not living with HIV, designed to assess HIV incidence and burden of other health outcomes. Eligibility for the cohort included being ≥age 18 years, speaking English or Spanish, residing in one of the eligible study cities, not living with HIV (based on laboratory confirmation), and identifying as a transgender woman, woman, or with a feminine gender identity, and being assigned a male sex at birth. Between March 2018–August 2020, 1312 transgender women were enrolled using two modes: a site-based technology-enhanced mode across six U.S. sites (Atlanta, GA, Baltimore, MD, Boston, MA, Miami, FL, New York, NY, Washington, DC) or exclusively digital mode geotargeted to 72 U.S. Eastern and Southern cities matched to the six site-based locations based on population demographics and size. Participants completed a baseline sociobehavioral assessment and HIV/STI testing and were followed for 24–48 months. Detailed study protocols have been published elsewhere [31,32].

Between February and November 2022, we conducted in-depth qualitative interviews with 27 Latina transgender women who participated in the LITE Study. Eligibility for the qualitative nested substudy were: (1) being enrolled in the LITE Study, (2) agreeing to be recontacted for future research, (3) identifying as Latina or having been born in a Latin American country. Interview participants were purposively sampled [33] from the 196 Latina transgender women in the LITE Study who met these criteria and responded to PrEP use measures at the baseline survey. This pool of preliminarily eligible participants was then stratified for sampling based on four a priori selected PrEP exposure groups to ensure a range of PrEP perspectives among participants: PrEP naïve—those who have never taken PrEP before and were not eligible to do so, PrEP eligible and not user—those who were eligible to take PrEP but had not started, current PrEP user–those who had taken PrEP within the last 30 days, and previous PrEP user–those who stopped taking PrEP more than 30 days before. Eligible individuals were invited to participate via text message or email (depending on contact preference) and asked to contact the study team if interested. Following an informed consent process, interviews were conducted online via Zoom in either English or Spanish by two bilingual interviewers (RAR and GV). Twenty-three interviews were conducted in English, and four in Spanish. Interviews lasted 60–90 min and were audio-recorded. All study procedures were reviewed and approved by the Institutional Review Board at Johns Hopkins University (protocol #IRB00142429).

### 2.2. Interview Guide

A semi-structured interview guide was developed collaboratively with the research team and community members. The guide included the following domains: Self-identity and context; HIV research engagement; experiences, perceptions, and preference related to PrEP; and Closing (see Supplementary Material File S1).

### 2.3. Data Analysis

Interviews were transcribed using Zoom’s automatic transcription feature as they were conducted. Members of the research team reviewed and de-identified the transcripts, checked them for accuracy, and cleaned them after each interview. Qualitative analyses were iterative in nature, employed a mix of inductive and deductive approaches, and incorporated weekly debriefing discussions held between the two interviewers (RAR and GV) as data collection progressed. An initial set of codes was developed based on the domains of the interview guide described above and informed by the Modified Social Ecological Model [17,18] and Intersectionality Framework [20,21,30]. Following each interview, field notes were recorded by both interviewers using a standardized template. The template included the initial set of codes, emerging themes, and preliminary findings, and guided debriefing discussions. A coding template was initially developed using the interview guide sections. This template was then modified by applying the study’s theoretical frameworks to identify themes related to individual, interpersonal, and structural levels and intersectionality. Deidentified transcripts were coded independently by two coders using Excel and consensus was achieved through ongoing discussions between coders and research team members. As additional themes emerged, these were added to the codebook and applied to transcripts. Interviews continued until saturation or redundancy of themes was achieved [34]. Crabtree and Miller’s five-step approach to qualitative analysis and interpretation was used that included describing, organizing, connecting, corroborating, and representing [35]. Summaries of major themes were developed across the four a priori selected PrEP exposure groups. Corroborating included discussing preliminary findings with the broader research team and community members.

### 2.4. Positionality and Reflexivity Statement

In order to address the unique needs of Latina transgender women, the team was led by Latinx/e transgender researchers with both lived experience and years of professional experience in transgender health research. The first, second, and fifth authors are transgender. The first and second authors are of Ecuadorian and Dominican backgrounds, born in Latin America and the U.S., respectively, and fluent in Spanish. The first author has a doctoral degree in Clinical Psychology, the second author has an associate degree in Business, the third author has a doctoral degree in International Public Health, the fourth author has a doctoral degree in Epidemiology, and the fifth author has a doctoral degree in Social and Psychiatric Epidemiology. The first author designed the nested study, and the first and second authors both conducted qualitative interviews in English and Spanish, allowing participants to select the language they were more comfortable speaking. Additionally, their backgrounds influenced the coding and interpretation of the interviews that required a nuanced understanding of acculturation processes among Latina transgender women moving from their home countries to the U.S. The process of writing, interpreting results, and implications involved the whole team in a collaborative process that included reflection, peer checking, questioning bias and assumptions, and revisiting the literature.

## 3. Results

### 3.1. Sample Characteristics

The average age of participants was 32.3 years old (range 18–70). Most Latina transgender women in the study identified as Multiracial (n = 14). The majority of participants were in the U.S., including Puerto Rico (n = 18). Participants originated across Latin America, with the largest number from Puerto Rico (n = 9). Most participants were U.S. citizens (n = 20). About half of the participants were unemployed (n = 14), with a large number of participants reporting food insecurity (n = 12). Similarly, a large number of participants (n = 12) reported an income below the 2018 federal poverty line (participants’ gross monthly household income and size were used to dichotomize this variable equating to an annual income of $12,140 for individuals living alone) [36]. Participants reported their current residence in many Eastern and Southern U.S. states, though the largest numbers were residing in New York (n = 9), Boston (n = 6), and Miami (n = 5). Most interviews were conducted in English (n = 23). Participants also had a relatively even distribution across PrEP exposure categories. Table 1 displays participant characteristics.

**Figure 1:**
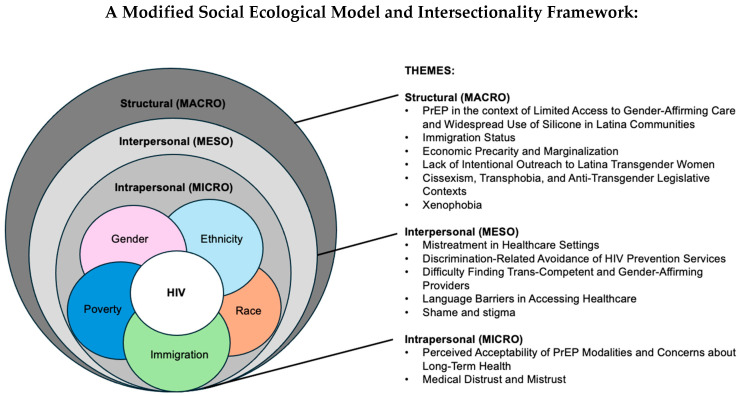
Multilevel and Intersectional Factors Identified by Latina Transgender Women in Qualitative Interviews.

The multilevel and intersectional themes from participants are displayed in Figure 1. Each theme is described in detail below.

### 3.2. Intrapersonal (MICRO) Level

#### 3.2.1. Perceived Acceptability of PrEP Modalities and Concerns About Long-Term Health

PrEP modalities were discussed with participants, including currently approved methods (daily oral pill, injectables) and future methods (implants, infusions, gel). The most commonly endorsed PrEP modality was an implant. One participant explained why they preferred the implant among other choices: “I would have to say implants. I feel they would take the least amount of maintenance” (20 yo, Black, Dominican Republic). Injection administration and topical gel applications followed in preference. The remaining options were selected by one participant each: pills, antibodies, IV fluids, whichever last longer, any of them, and neither.

Regardless of PrEP modality, side effects and negative long-term health effects were described as a deterrent to PrEP use by multiple participants. One participant, who mentioned that they would not take any PrEP modality, said: “to be honest, I would have preferred neither of them, because they all can kill you. They all cause liver disease” (18 yo, Multiracial, Puerto Rico). Latina transgender women were often unsure whether PrEP medications would actually be beneficial to their health, concerns which were compounded by other access challenges like affordability. When asked about the maximum they would be willing to pay for PrEP, one participant stated, “Absolutely nothing, if it is going to cause all those side effects” (18 yo, Multiracial, Puerto Rico). Although side effects were commonly discussed, fear of interactions with hormones were not mentioned as a barrier.

#### 3.2.2. Medical Distrust and Mistrust

Latina transgender women reported medical mistrust in general and while accessing PrEP. Latina transgender women felt dishonest intentions from providers prescribing medications like PrEP. One Latina transgender women described her experience and lacking trust in her provider: “He’s like, why are you not on PrEP? And I was like well, one I’m celibate and I don’t really think that it’s safe, you know. I have a friend that lost a tooth, like lost a lot of bone density and shit like that from taking PrEP. I feel like the way that they push it, like I get PrEP is good, HIV prevention is good, you know. But I feel like they don’t give us enough information. He’s like it’s totally safe. Just take it. Just trust me, you know, and I’m like, Why the fuck would I trust you? You’re not giving me a satisfactory answer, like trust me is not a satisfactory answer. I feel like that was an example of my concerns being dismissed, because I was just asking about, how long has it been studied for? How many people has it been tested on like, what are the potential side effects, you know? I feel like that was just kind of like brushed off” (21 yo, White, Puerto Rico).

Latina transgender women who had never accessed PrEP also described feeling medical mistrust in deciding whether to access PrEP, in some cases referencing racist and unethical research practices historically and currently. One participant described how Latina transgender women were “being used as guinea pigs because they’re not as valued, they’re being used to test things that will be for the White people. Thanks a lot Tuskegee” (37 yo, Multiracial, U.S. born from Mexican and Ecuadorian heritage). Several participants also mentioned medical distrust in relation to PrEP marketing by pharmaceutical companies: “I can’t deal with the side effects and don’t feel like the marketing of PrEP has been entirely honest” (21 yo, White, Puerto Rico).

### 3.3. Interpersonal (MESO) Level

#### 3.3.1. Mistreatment in Healthcare Settings

Mistreatment in healthcare systems was a common theme. Almost all participants reported experiencing mistreatment in healthcare setting due to being Latina transgender women. In the words of one participant, “You just get categorized as soon as you walk in the door” (23 yo, Multiracial, U.S. born, heritage not specified). Latina transgender women commonly reported being negatively perceived in healthcare settings, resulting in poor treatment. Latina transgender women often feared providers would turn them down for essential services simply because of who they are. One Latina transgender women said: “Pray to God, that maybe something might not happen to me. You know I have seen that happen before” (24 yo, Multiracial, Puerto Rico).

This theme also encompassed healthcare stereotypes and perceptions of Latina transgender women as “difficult patients” in accessing healthcare: “When you are trying to access the care you need and you are being told that you are asking for too much or that you are wrong and you are making things up and you are being inappropriate, when you’re literally just advocating for yourself as a patient that shit is traumatizing… it makes it terrifying to look for other medical help and it’s discouraging when you have an experience like that—repeated experiences—you start to think that it’s going to be like that all the time” (21 yo, White, Puerto Rico). Prior experiences of mistreatment and fear of future mistreatment impacted access to healthcare for Latina transgender women.

Latina transgender women in our study also faced mistreatment when trying to access HIV services. Mistreatment at the intersection of gender and race/ethnicity was exacerbated for Latina transgender women experiencing language barriers due to English language proficiency. One participant described these challenges in accessing HIV prevention services: “Depending on the strength in language, providers are not patient, they may overlook your care, and they will not give you time, appointments are very quick, or you have to wait longer. Some providers used my dead-name, that compromises your safety. There are a lot of safety factors when going to the clinic and sometimes doctors don’t even know how to treat you” (29 yo, Black, U.S. from Costa Rican/Panamanian heritage). The intersectional mistreatment due to both being a transgender woman and having communication difficulties due to language barriers, resulted in suboptimal care. Latina transgender women also described being careful and selective with where they accessed HIV prevention services due to the fear of being discriminated against.

#### 3.3.2. Discrimination-Related Avoidance of HIV Prevention Services

More than half of the Latina transgender women avoided accessing HIV and PrEP services because they felt that they might be treated poorly and feared discrimination. In the words of one participant: “They (Latina transgender women) are scared to go to a clinic, say that they need help, when they need some medication or treatment, or something, because of the fear that they might feel that they might be rejected” (40 yo, Multiracial, Mexico). Another participant described an experience of feeling judged and discriminated against by a White trans woman while trying to access services: “One time I went to get a regular HIV testing… When I went in to get screening, it was another White trans woman. When I told her what I was coming in for she made me feel disgusting. She made me feel like a lot of Latinas don’t wrap it up, which is not true. She should be let go, she should not make them feel judged” (26 yo, Multiracial, Puerto Rico). These previous experiences of discrimination, including from within transgender communities by White transgender women delivering HIV prevention services, were highlighted as contributing to healthcare avoidance, uptake of HIV services and PrEP, and quality of care received.

#### 3.3.3. Difficulty Finding Trans-Competent and Gender-Affirming Providers

Latina transgender women reported avoiding HIV prevention services, including PrEP, at medical institutions and locations that they heard did not have sufficient knowledge in how to care for Latina transgender women. Latina transgender women emphasized the importance of and challenges with identifying trans-friendly and gender-affirming providers for healthcare in general and for HIV prevention services specifically. When asked about challenges accessing HIV prevention, PrEP, and healthcare, one participant noted: “First one that comes to mind is finding a doctor that is, transgender friendly, that you feel comfortable working with that would give you it [PrEP]. Also, you know, access to it like, where is this accessible? Is it available in a lot of rural areas? A lot of people that are in rural areas might not be able to find, you know, a clinic close to them that would provide it” (22 yo, White, U.S. from Nicaraguan/Panamanian heritage). Geographic location was highlighted in discussing issues finding trans-friendly providers, including distance from urban centers for those who resided in a catchment area proximal to a city but not inside the city limits or states that are not consider trans-friendly. For instance, a participant living in Florida noted: “There’s not much for trans people, no help, no assistance. There’s nothing here. Some other girls they just leave, they move out they go to other states where they feel a little bit more comfortable. Here is just the fact that there is no help” (46 yo, White, Puerto Rico).

### 3.4. Language Barriers in Accessing Healthcare

Latina transgender women perceived language as a challenge that they faced in accessing healthcare. Language barriers were discussed in relation to language comprehension for Latina transgender women immigrant communities who were marginalized due to being both an immigrant and transgender woman. One participant said, “It’s challenging for Latina transgender women students who immigrate here and have to learn English on top of continuing with schooling, it can definitely be a huge struggle. I think adding on top of that experience, you’re also trans, that would just compound the feelings of isolation and struggle to find you afloat in this new area” (21 yo, White, U.S. heritage not specified). Latina transgender women who experienced language barriers also described language in accessing healthcare. One participant stated, “When you don’t have the English capability, the language capability that allows you understand more of the North American system, it is as if you are lost because there are many things that we don’t know have to manage it, that make it harder, you make an effort when you seek help because we seek help more than others but we stumble with the language” (24 yo, Multiracial, Dominican Republic).

This participant referred to providers not giving Latina transgender women the proper care and support needed because Latina transgender women could not communicate “in an eloquent way” with providers due to language barriers. The lack of Spanish-speaking providers in healthcare contexts was also discussed by participants as a barrier to accessing care.

#### Shame and Stigma

Shame and stigma arose among Latina transgender women when discussing access to PrEP services. Latina transgender women often described feeling judged and labeled as promiscuous in relation to their sexuality and sexual behaviors. One participant explained how terminology sometimes deterred Latina transgender women from accessing services like PrEP: “When it gets morally judged, when people say it’s high-risk behavior, I mean yeah it is, but I think some of those terms can come off in a way that probably feels judgmental and discouraging to people that choose things like that. So again, they may feel judged and maybe feel like I can’t be honest about certain things, and it can make it harder for Latina transgender women to get the treatment that they need and adhere to the treatment” (21 yo, White, Puerto Rico). Stigma was especially highlighted as playing a part in why Latina transgender women may not access PrEP, particularly for Latina transgender women engaging in survival sex work: “I feel there is a stigma that HIV comes from the LGBT community, and that is not true. People might associate that [HIV] with [Latina transgender women]. That was her life and that’s what she did, so that comes with consequences. People [Latina transgender women] might not want to go over there and be judged” (24 yo, Multiracial, Puerto Rico). This participant described internalized stigma as a driver for why Latina transgender women do not access PrEP services, especially for those who are sex workers, and shame resulting from internalizing societal stigma that HIV originates from the transgender community and that it is also a moral consequence of their work.

Latina transgender women also mentioned stigma from sexual partners as a barrier to PrEP access: “It still can be an ongoing effort to be able to get the prescribed medications regularly and therefore be able to take them rightly… there is continual access [difficulty] and potential pushback from partners as well. Some of us face partners [who] treat us as though we’re more at their mercy” (37 yo, Multiracial, U.S. from Mexican/Ecuadorian heritage). Stigma from partners, and potential violence, were involved in Latina transgender women decision-making about HIV prevention services.

### 3.5. Structural (MACRO) Level

PrEP in the context of Limited Access to Gender-Affirming Care and Widespread Use of Silicone in Latina Transgender Communities

Participants commented on the limited access to gender-affirming care through medical institutions such as hospitals, which multiple Latina transgender women reported led to obtaining hormones from outside of medical settings and to widespread use of silicone for feminization and body enhancement among Latina transgender women. Some Latina transgender women discussed injectable PrEP uptake as a challenge for those who had injected silicone or other foreign material (“fillers”) into areas where injectable PrEP may need to be administered, such as the buttocks. One participant described this: “Some trans women are going to have to make a decision [whether or not to take PrEP] based on whether they have silicone in their buttocks. A lot of Latina transgender women have a lot of silicone in their buttocks, and they can’t inject medication into that area” (37 yo, White, El Salvador). Another participant felt that Latina transgender women were “forgotten” and structurally excluded from taking LAI PrEP due to silicone use: “We wasn’t the target as Latina transgender women. They are forgetting one thing, most of us have silicone implants so that’s quite difficult, they cannot inject the PrEP injection on top of the silicone implants. I believe we wasn’t the target they forget about us Latina transgender women” (43 yo, White, Honduras). Although several Latina transgender women were open to taking LAI PrEP and were comfortable having medication like hormones and other gender enhancements administered in the buttocks area, those who had injected silicone or biopolymers into that area had heard from providers they could not use it.

### 3.6. Immigration Status

Latina transgender women in this sample who were born in another country described unique challenges accessing healthcare in the U.S. related to structural exclusion. One participant described how Latina transgender women who immigrate into the country are not getting access to the resources that they need: “They don’t provide them [immigrant Latina transgender women] the correct healthcare, they’re rotting in these prisons, and they’re not getting the necessary healthcare, and they should just have resources in general for Latina transgender women. They come into the country, and there’s like no resources to explain to them like what’s here? What you have to do, like what resources about healthcare and every healthcare should explain to you about for every kind of risk” (24 yo, Multiracial, U.S. from Puerto Rican/Ecuadorian heritage). Another participant explained how being transgender increases the difficulties when immigrating: “We are transgender, if for a normal person immigration is difficult, you can imagine for a transgender person, it is double the difficulty because we get harassed… bullied, we get treated differently… because we look different, because of our appearance, because of our differences, and they make fun of us” (40 yo, Multiracial, Mexico).

In their countries of origin, Latina transgender women commonly reported prior experiences of mistreatment, driven by transphobia, and had limited access to healthcare. Latina transgender women who had immigrated reported continuing to avoid healthcare in the U.S. due to the mistreatment they had faced in their countries of origin: “We [immigrant Latina transgender women] believe that it would be the same as over there [country of origin], obviously it could be like that with occasion, but here [in the U.S.] if you complain they listen. It’s super different. My colleagues don’t understand that here in America it’s not the same [as their country of origin]. That is why it’s very likely that Latina transgender women [who immigrate] don’t access healthcare” (37 yo, White, El Salvador). The participant described how Latina transgender women who immigrated to the U.S. had preconceived thoughts that they would receive the same mistreatment or lack of quality care as in their native countries when trying to access services in the U.S.

Fear of deportation led many Latina transgender women to not access basic health necessities, and to not vocalize their needs because they felt like their freedom was at risk and they could end up being deported. In some cases, their gender transition was delayed as a result of fear of deportation, as shared by one participant: “Immigration was a big challenge for me, and one of the reasons I didn’t transition until later on. I am a Dreamer under the Deferred Action for Childhood Arrivals [DACA], and my family came legally and applied for asylum, and we were denied over technicalities, and we overstayed. So that meant that for my high school and beginning of my college career I had no papers, so I had to apply as an international student and wasn’t able to get legal jobs. So, at that time, I stayed away from trying to transition or even thinking about it because my family when I did try to come out said that would be used against me and that I would be deported. So, it was used directly against me. If I came out and allowed myself to be trans I would be deported. So that kept me in the closet for a long time… Once DACA was passed and I did receive a work permit, it opened up a lot of opportunities to go back to school, to get health insurance, and I was able to look up for community resources…. But I wasn’t able to get on sliding scale until I had insurance, until I had a paystub, until I had papers. It was an uphill climb even knowing where the resources were” (27, Multiracial, Venezuelan). Fears of deportation and related U.S. policy challenges were frequently discussed by participants born in another country.

### 3.7. Economic Precarity and Marginalization

The costs of PrEP (both the medication and ancillary costs) were a barrier to PrEP uptake for Latina transgender women. Among the 27 participants in our study, only four were aware of information regarding coverage or copay when trying to access PrEP medications. Affordability and lack of knowledge about PrEP financial coverage or resources were the biggest challenges that Latina transgender women encountered in accessing PrEP. We asked participants the maximum amount that Latina transgender women, including themselves, would be willing to pay for PrEP. The majority answered that there should be no cost. As one participant stated, “Free. I’m struggling to keep up… I don’t even have $5 in my savings” (45 yo, White, Puerto Rico). Another participant said, “It should be free, we shouldn’t be paying for any medication” (31 yo, Multiracial, U.S. from Mexican heritage). In the context of economic survival, PrEP cost prevented uptake for Participants.

Participants reported that there were misconceptions within the Latina transgender women community about qualifying for PrEP and accessing assistance with PrEP coverage. One Latina transgender women described being unable to afford PrEP medication even if it had minimal cost for copays: “One of the main reasons is that I just couldn’t afford it, even if it was sometimes $3.50 copays. Sweetheart, I get $876 a month. That’s nothing. The same day that money comes, it goes to pay everything, so sometimes I don’t have the money [for PrEP]” (45 yo, White, Puerto Rico). None of the participants mentioned or had knowledge about financial assistance resources for PrEP medication. Latina transgender women understood the importance of PrEP, but the burden of affordability was a factor as to why they were unable to access it or stay on the medication if they were already on PrEP.

Employment was consistently raised as a structural issue by Latina transgender women. Many participants described that Latina transgender women were pushed into sex work due to challenges with employment: “They pushed us to think we can only be escorts” (23 yo, Multiracial, U.S. heritage not specified). Latina transgender women who were able to get employment often discussed facing discrimination, being sexualized, and not being able to grow at their place of work, getting fewer opportunities for advancement compared to their cisgender colleagues. Many Latina transgender women, particularly if not employed, had a challenging time gaining access to insurance. One participant stated: “A big struggle, if you can’t maintain regular work, then how are you supposed to maintain regular health insurance, which is needed to get medication?…. You need a good insurance that’ll cover your medications or else you’re paying like up the walls” (21 yo, White, U.S. heritage not specified). For Latina transgender women immigrants who were undocumented and could not access federally funded insurance or subsidies, insurance-related obstacles to PrEP access were particularly evident.

### 3.8. Lack of Intentional Outreach to Latina Transgender Women

Outreach was discussed as an important aspect of healthcare access for Latina transgender women. One participant described the lack of outreach to Latina transgender women as impacting access to HIV prevention: “Here, no one is really trying to bring you into that access or reaching out to you, making sure that you get those things [HIV prevention services]. It’s more of a secret. It could even be illegal” (21 yo, White, U.S. heritage not specified). Latina transgender women from Atlanta and Miami especially mentioned unmet outreach needs: “People are not being taught about PrEP and how to take care of themselves, especially in South Florida and Atlanta. There isn’t enough education on prevention” (24 yo, Multiracial, U.S. from Puerto Rican/Ecuadorian heritage).

Some Latina transgender women resided in states facing possible legislative bans when trying to access gender-affirming services, particularly those in Florida, and mentioned the need for more intensive outreach. Immigrant Latina transgender women in the U.S. were identified as a group in need of focused outreach efforts: “Immigrants are not getting access to knowledge on medication and prevention resources like PrEP” (24 yo, Multiracial, U.S. from Puerto Rican/Ecuadorian heritage). Structural barriers, including lack of outreach to Latina transgender women communities, varied depending on the geographic location where Latina transgender women resided. Many of the Latina transgender women who were previous PrEP users resided in the Northern U.S. and described lack of support for continuing PrEP use and adherence.

### 3.9. Cissexism, Transphobia, and Anti-Transgender Legislative Contexts

Transphobia and the contemporary legislative context of anti-trans bills in the U.S. were brought up by many participants. Anti-trans bills were viewed as a form of political attack and made Latina transgender women feel unsafe, targeted, and insecure in their healthcare rights: “Anti-trans bills are giving permission for people to be violent. Justice system does not enforce laws to stop, and they allow it. Healthcare, we just got it back after Trump, and now it feels is being taken away again” (29 yo, Black, U.S. from Costa Rican/Panamanian heritage). Latina transgender women emphasized that their access to healthcare was being jeopardized. Participants living in the South, particularly states being targeted by anti-trans legislation, often discussed feeling unsafe: “Trump impacted Latinx in general, but now trans people are being attacked on social media politically, and it’s the biggest thing right now because we are becoming so visible it is creating uproar among racist transphobes. POC are the ones dealing with it the most, the most attacked. I became a gun owner for that reason, I purchased a gun to feel safe. I will not walk in a country and feel unsafe, especially for being who I am. Driving from Georgia to Alabama I was shot at, and that changed my whole perspective” (24 yo, Multiracial, U.S. Puerto Rican/Ecuadorian heritage). The anti-trans policies were described as particularly salient for Latina transgender women communities, given the intersection of gender and ethno-racial identities, and therefore transphobia and racism, in their lived experiences.

### 3.10. Xenophobia

Xenophobia was also described as a major structural barrier impeding Latina transgender women’s access to healthcare and intensified feelings of being unsafe. One Latina transgender women participant from Puerto Rico described her experience: “I’ve been told go back to my country, so people don’t understand PR (Puerto Rico) is part of U.S. I’ve been called a SPIC; people are ignorant” (26 yo, Multiracial, Puerto Rico). Xenophobia towards immigrant Latina transgender women was also highlighted by this participant: “When you don’t have papers, well, they treat you worse because who is going to provide you with care, who is going to save you, they do with you what they want and who will defend you. I can’t go to the police without papers or to file reports because that will get you in trouble” (24 yo, Multiracial, Dominican Republic).

Moreover, Latina transgender women in this study described the double jeopardy they experienced, the combined negative effects of multiple systems of oppression, as they face both xenophobia and transphobia. As one participant stated: “When you try to come from another country into the U.S. and try to get legal documents, legal paperwork to appropriately identify you as a woman, as a trans woman in this country is very difficult especially because the way this country is, they don’t want immigrants coming into this country period. When somebody comes into this country and you are trans on top of that is like you got two X’s on you already, two strikes on you. It is very difficult to even be respected when you try to immigrate into this country as a trans person because trans people are rarely respected in this country… Trans people are rarely respected in this country, so you can only imagine what it would be like for a trans immigrant trying to get… an immigration card and become a citizen in this country” (25 yo, Multiracial, Dominican Republic).

## 4. Discussion

This nested qualitative substudy of Latina transgender women in the Eastern and Southern U.S. identified multiple intersectional intrapersonal, interpersonal, and structural barriers and facilitators to PrEP uptake and persistence. Corroborating prior research on barriers and facilitators to PrEP use among transgender women [22,23], Latina transgender women in this sample identified medical distrust and mistrust, mistreatment in healthcare, avoidance of services due to discrimination, difficulty finding trans-competent and gender-affirming providers, and economic barriers as presenting challenges. However, guided by a Modified Social Ecological Model [17,18] and Intersectionality Framework [20,21,30], we also found that Latina transgender women faced unique issues due to intersecting oppression as both Latinas and transgender women, as well as by immigration status, whether perceived or real. Extending prior research on PrEP [11,13,15,29] and healthcare access [24] among Latina transgender women, our study identified additional considerations for interventional research and programming with Latina transgender women, including language barriers and lack of Spanish-speaking providers, immigration-related concerns including fear of deportation for those who were undocumented, xenophobia, stigma and stereotypes related to being Latina transgender women, lack of intentional outreach efforts, and limited access to gender-affirming care alongside common use of silicone fillers that limit uptake of LAI-PrEP. Cissexism and transphobia were also highlighted, including the contemporary anti-trans legislative environment, likely due to this study being conducted in early 2022, coinciding with an unprecedent increase in the introduction of anti-trans bills in the U.S. when went from 32 bills in 2019 to 174 bills in 2022—a 444% increase over a 3-year period. A number that rose to an all-time high in 2025, with 854 anti-trans bills [37]. In 2025, the Trump administration has issued multiple executive orders directly attacking the transgender population in the U.S. that includes mandates to define gender in terms of a male-female sex binary, preventing gender-affirming care for people under 19, banning transgender people from sports and the military, among others [38]. More than ever, findings can be used to inform tailored HIV prevention efforts that address the modifiable intersectional intrapersonal, interpersonal, and structural level factors identified by Latina transgender women in this study for PrEP uptake and service delivery.

Latina transgender women immigrants in our study reported unique challenges relating to PrEP access and uptake that non-immigrant participants did not discuss, which extends prior work Latina transgender women immigrant communities’ experiences navigating general healthcare systems [24]. Language barriers were a prominent theme, highlighting the need for more Spanish-speaking providers and healthcare staff. Another challenge related to the fear of deportation and perceived risk of involuntary removal due to violation of immigration laws, which was a chilling effect observed for many Latinx patients in the U.S. [39]. For Latina transgender women who are undocumented, avoidance of healthcare was high with migration policies negatively affecting access to PrEP care. Another immigration-related challenge was insurance. Latina transgender women immigrants who are naturalized citizens or permanent residents are eligible to receive federally funded health insurance coverage under the ACA (Affordable Care Act) with stipulations, but those who are undocumented are not eligible to receive federally funded coverage [40]. This makes PrEP inaccessible to many Latina transgender women who could benefit. State-funded programs and private pharmaceutical companies are ways in which Latina transgender women immigrants can acquire PrEP; however, Latina transgender women immigrants are not being fully informed on how to get coverage through state-funded or other programs that assist with PrEP coverage. Through our study we saw how compounding challenges, like difficulty being able to communicate with providers, fear of being deported, and lack of accessible insurance, accumulate and make it hard for Latina transgender women immigrants to access PrEP, underscoring the ways that migration policies are barriers to PrEP for Latina transgender women who immigrate, which are barriers that have only worsened during the second Trump administration with increased inhumane mass detention and deportation efforts [41].

In this study, Latina transgender women were open to different PrEP modalities without clear consensus. Acceptability was highest for the PrEP implant application, followed by injection and gel application. These preferences for PrEP modalities within Latina transgender women in our study may be attributable to being familiar and comfortable with gender-affirming injections and topical hormone gels. Latina transgender women were open to different modalities if it was convenient and easy for their desired need and circumstance. Latina transgender women faced many compounding challenges in their lives and preferred a modality that would make taking PrEP easy and minimize challenges. This differentiated from other studies focused on Latino MSM communities, which found a preference for injectable PrEP (42%), followed by pill form (35%) [42]. The daily oral pill form was a modality that Latina transgender women in our study were less in favor of due to difficulty adhering to taking a pill every day. For Latina transgender women to make adequate informed decisions in regard to choosing PrEP and the right PrEP modality for them, clearer campaigns that detail specifics of each PrEP modality need to be made more readily available to facilitate PrEP access. Medication modalities need to cater to the specific needs of Latina transgender women and differentiate what modality fits each person best. Providing multiple PrEP modalities serves as a facilitator by offering Latina transgender women options to choose what best fits their needs. Social barriers like stigma influence PrEP uptake and need to be addressed by PrEP campaigns for Latina transgender women. Consistent with prior research [13], Latina transgender women in our study reported shame and stigma in the context of healthcare generally, and in accessing HIV prevention services specifically, fueled by stereotypes about Latina transgender women related to promiscuity, moralizing of “risky” sexual behaviors, and HIV, which impacted PrEP engagement. Within our study, we saw that Latina transgender women had high awareness of PrEP but low uptake or not taking the medication at all, which corroborates prior studies that have been published on unmet needs for PrEP [7,10].

Corroborating prior qualitative research with Latina transgender women [11,12,29], participants in the current study identified competing priorities other than HIV, including economic survival and gender-affirming care, as barriers to PrEP access and uptake. A large number of participants reported being unemployed, facing food insecurity, and living below the federal poverty line. As such, Latina transgender women in our study described not being able to pay for PrEP if costs were not fully covered because they were prioritizing other competing needs, including the costs that came along with other medications and with gender-affirming care. All participants mentioned that they were not able to afford PrEP if it was not covered by insurance and were exclusively dependent on insurance coverage to whether or not they would be able to access and adhere to PrEP medication. Only four were aware of PrEP medication coverage programs or financial assistance programs for PrEP. This is consistent with prior research showing substantial PrEP knowledge gaps within Latina transgender women communities which contribute to low medication uptake for this community [27]. Healthcare institutions and healthcare professionals need to provide Latina transgender women with information, financial assistance program options, and coverage resources to facilitate and inform PrEP decision-making and access.

Prior research has shown that medically unsupervised gender-affirming care and procedures are common among Latina transgender women due to socioeconomic vulnerabilities and lack of access to appropriate healthcare [43,44]. One study of 205 Latina transgender women found that 68% reported non-prescribed hormone use and that undocumented immigration status was associated with a fivefold higher odds of using non-prescribed hormones [43]. In a study of 631 adult transgender women in the San Francisco Bay area, filler usage was highest for Latina transgender women compared with White transgender women (21% vs. 4%; *p* < 0.001), and for those with a history of being undocumented vs not (32% undocumented, 16% never undocumented, 6% born in the US; *p* < 0.0001) [44]. In our study, Latina transgender women prioritized hormonal treatments over prevention medications like PrEP due to cost. Given prior research demonstrating that medical gender affirmation is associated with PrEP uptake in transgender women [45,46], integration of hormones and gender-related care represents an important PrEP facilitator in future interventions. Furthermore, study participants also highlighted the common use of “fillers” such as silicone in the buttocks and gluteal areas for gender enhancement by Latina transgender women and identified this as a barrier to injectable PrEP uptake. PrEP injections are not currently approved for those who have silicone injected into their buttocks and gluteal areas because it has not been widely researched. Additional research is needed on LAI PrEP and silicone fillers among transgender women, especially given potential interest in LAI-PrEP. A recent study of perceived acceptability of LAI PrEP among 867 U.S. transgender women found that 47% were “somewhat” to “very interested” in LAI PrEP, with no statistically significant differences in perceived acceptability by ethnicity for Latina transgender women [47]. Education is also necessary for Latina transgender women, as well as healthcare providers, including the potential for utilizing other sites for injectable PrEP agents, such as the deltoid muscle in the arm rather than the gluteal muscle.

Our findings are subject to several limitations. In qualitative research, multiple shared identities between interviewers and interviewees can present both benefits and challenges to data quality. For example, it is possible that interviewers may make assumptions about interviewees’ experiences, and there may be heightened risk for confirmation bias. However, these similarities also carry significant benefits, such as enhanced rapport, deeper understanding of overlapping shared experiences, and increased empathy, which can foster open and honest conversations. In our study, we engaged in reflexivity by critically reflecting on our own positionality and biases to ensure a diverse range of perspectives. Additionally, this was a nested qualitative substudy that drew participants from an existing longitudinal cohort study in the Eastern and Southern U.S.; thus, findings may not be generalizable to Latina transgender women in other parts of the country or who may not choose to participate in research. A strength of our approach is that we were able to leverage quantitative survey data from an ongoing cohort of transgender women to glean in-depth insights into the unique contexts of PrEP uptake, unmet needs, and barriers to PrEP service delivery for Latina transgender women for future interventions, programming, and services. The six sites that recruited and enrolled participants either provided clinical services, including HIV prevention and gender-affirming care services, or had close partnerships with trans-serving organizations. Therefore, Latina transgender women in this study may have been more knowledgeable about PrEP than Latina transgender women not drawn from these settings; replication is needed with Latina transgender women not as connected to clinical care.

## 5. Conclusions

Transgender women are a priority population for HIV prevention efforts in the U.S. [3,4]. Given the documented high HIV burden among Latina transgender women [1], tailored efforts are needed to optimize uptake of available prevention methods, including PrEP. This nested qualitative substudy of Latina transgender women in the Eastern and Southern U.S. identified multiple intersectional intrapersonal, interpersonal, and structural barriers and facilitators to PrEP uptake and persistence that are amenable to interventions, some common to all transgender women, and others unique to Latina transgender women. Culturally tailored HIV prevention efforts are necessary. There is a need to address intrapersonal barriers such as medical distrust and mistrust to increase PrEP uptake. At the interpersonal level, there is a need to increase interaction with trans-competent providers and culturally responsive staff, enhance a sense of safety in accessing HIV prevention services, and offer HIV prevention outreach and PrEP services delivered in Spanish. In the context of transphobia, xenophobia and contemporary anti-transgender and anti-immigrant legislation climate, at the structural level there is a need to increase state coverage of HIV prevention services, primarily PrEP, to undocumented and uninsured Latina transgender women. Policy changes are needed to ensure equitable access to PrEP and that Latina transgender women can achieve their HIV prevention needs. Prior interventional research has demonstrated that creating trusted spaces, providing peer navigation for services, offering peer support, and integrating gender-affirming care facilitate and garner high acceptability in delivering PrEP services for Latina transgender women [27,48]. Interventions such as these and others that address the multiple intersectional intrapersonal, interpersonal, and structural barriers and facilitators to PrEP uptake identified by participants in this study represent an important next step.

## Figures and Tables

**Table 1:** Participant Demographics.

Characteristic	Total = 27
Age (Mean)	32.3
	(range 18–70)
Race/Ethnicity	
Black	2
Multiracial	14
White	11
Place of Origin	
Cuba	1
Dominican Republic	3
Honduras	1
Mexico	2
Puerto Rico	9
El Salvador	1
U.S. born (Costa Rican/Panamanian heritage)	1
U.S. born (Cuban heritage)	1
U.S. born (Mexican heritage)	1
U.S. born (Mexican/Ecuadorian heritage)	1
U.S. born (Nicaraguan/Panamanian heritage)	1
U.S. born (Puerto Rican/Ecuadorian heritage)	1
U.S. born (heritage not specified)	3
Venezuela	1
Citizenship Status	
U.S. Citizen	20
Not a U.S. Citizen	7
Employment	
Full time	7
Part time	3
Unemployed	14
Prefer not to answer	3
Income	
Above the Federal Poverty Line	8
Below the Federal Poverty Line	12
Unknown	7
Food Insecurity	
Yes	12
No	15
Interview Language	
English	23
Spanish	4
Cohort Site	
Atlanta, GA	1
Boston, MA	5
Miami, FL	5
New York, NY	9
Washington DC	2
Online (Allenton, PA)	1
Online (Boston, MA)	1
Online (Chicago, IL)	2
Online (Tampa, FL)	1
PrEP Exposure	
PrEP Naïve	8
PrEP Eligible and Not User	5
Current PrEP User	6
Previous PrEP User	8

## Data Availability

The data presented in this study are available on request from the corresponding author. The data are not publicly available due to privacy and ethical restrictions.

## Supplementary Materials

The following supporting information can be downloaded at: https://www.mdpi.com/article/10.3390/ijerph22050659/s1, File S1: Interview Guide.

## References

[1] Becasen J.S., Denard C.L., Mullins M.M., Higa D.H., Sipe T.A. (2019). Estimating the Prevalence of HIV and Sexual Behaviors Among the US Transgender Population: A Systematic Review and Meta-Analysis, 2006–2017. Am. J. Public Health.

[2] Stutterheim S.E., van Dijk M., Wang H., Jonas K.J. (2021). The worldwide burden of HIV in transgender individuals: An updated systematic review and meta-analysis. PLoS ONE.

[3] Barry M.J., Nicholson W.K., Silverstein M., Chelmow D., Coker T.R., Davis E.M., Donahue K.E., Jaen C.R., Kubik M., U.S. Preventive Services Task Force (2023). Preexposure Prophylaxis to Prevent Acquisition of HIV: US Preventive Services Task Force Recommendation Statement. JAMA.

[4] Fauci A.S., Redfield R.R., Sigounas G., Weahkee M.D., Giroir B.P. (2019). Ending the HIV Epidemic: A Plan for the United States. JAMA.

[5] Lee K., Trujillo L., Olansky E., Robbins T., Brune C.A., Morris E., Finlayson T., Kanny D., Wejnert C., National HIV Behavioral Surveillance Among Transgender Women Study Group (2022). Factors Associated with Use of HIV Prevention and Health Care Among Transgender Women—Seven Urban Areas, 2019–2020. MMWR Morb. Mortal Wkly. Rep..

[6] Wirtz A.L., Humes E., Althoff K.N., Poteat T.C., Radix A., Mayer K.H., Schneider J.S., Haw J.S., Wawrzyniak A.J., Cannon C.M. (2023). HIV incidence and mortality in transgender women in the eastern and southern USA: A multisite cohort study. Lancet HIV.

[7] Centers for Disease Control and Prevention (CDC) (2021). HIV Infection, Risk, Prevention, and Testing Behaviors Among Transgender Women—National HIV Behavioral Surveillance, 7 U.S. Cities, 2019–2020 (HIV Surveillance Special Report 27).

[8] Grant R.M., Lama J.R., Anderson P.L., McMahan V., Liu A.Y., Vargas L., Goicochea P., Casapia M., Guanira-Carranza J.V., Ramirez-Cardich M.E. (2010). Preexposure chemoprophylaxis for HIV prevention in men who have sex with men. N. Engl. J. Med..

[9] Landovitz R.J., Donnell D., Clement M.E., Hanscom B., Cottle L., Coelho L., Cabello R., Chariyalertsak S., Dunne E.F., Frank I. (2021). Cabotegravir for HIV Prevention in Cisgender Men and Transgender Women. N. Engl. J. Med..

[10] Malone J., Reisner S.L., Cooney E.E., Poteat T., Cannon C.M., Schneider J.S., Radix A., Mayer K.H., Haw J.S., Althoff K.N. (2021). Perceived HIV Acquisition Risk and Low Uptake of PrEP Among a Cohort of Transgender Women With PrEP Indication in the Eastern and Southern United States. J. Acquir. Immune Defic. Syndr..

[11] Poteat T., Wirtz A., Malik M., Cooney E., Cannon C., Hardy W.D., Arrington-Sanders R., Lujan M., Yamanis T. (2019). A Gap Between Willingness and Uptake: Findings From Mixed Methods Research on HIV Prevention Among Black and Latina Transgender Women. J. Acquir. Immune Defic. Syndr..

[12] Nieto O., Fehrenbacher A.E., Cabral A., Landrian A., Brooks R.A. (2021). Barriers and motivators to pre-exposure prophylaxis uptake among Black and Latina transgender women in Los Angeles: Perspectives of current PrEP users. AIDS Care.

[13] Brooks R.A., Cabral A., Nieto O., Fehrenbacher A., Landrian A. (2019). Experiences of Pre-Exposure Prophylaxis Stigma, Social Support, and Information Dissemination Among Black and Latina Transgender Women Who Are Using Pre-Exposure Prophylaxis. Transgend. Health.

[14] Cooney E.E., Reisner S.L., Saleem H.T., Althoff K.N., Beckham S.W., Radix A., Cannon C.M., Schneider J.S., Haw J.S., Rodriguez A.E. (2022). Prevention-effective adherence trajectories among transgender women indicated for PrEP in the United States: A prospective cohort study. Ann. Epidemiol..

[15] Storholm E.D., Ogunbajo A., Nacht C.L., Opalo C., Horvath K.J., Lyman P., Flynn R., Reback C.J., Blumenthal J., Moore D.J. (2024). Facilitators of PrEP Persistence among Black and Latinx Transgender Women in a PrEP Demonstration Project in Southern California. Behav. Med..

[16] Deutsch M.B., Glidden D.V., Sevelius J., Keatley J., McMahan V., Guanira J., Kallas E.G., Chariyalertsak S., Grant R.M., iPrEx Investigators (2015). HIV pre-exposure prophylaxis in transgender women: A subgroup analysis of the iPrEx trial. Lancet HIV.

[17] Baral S., Logie C.H., Grosso A., Wirtz A.L., Beyrer C. (2013). Modified social ecological model: A tool to guide the assessment of the risks and risk contexts of HIV epidemics. BMC Public Health.

[18] Poteat T., Scheim A., Xavier J., Reisner S., Baral S. (2016). Global Epidemiology of HIV Infection and Related Syndemics Affecting Transgender People. J. Acquir. Immune Defic. Syndr..

[19] White Hughto J.M., Reisner S.L., Pachankis J.E. (2015). Transgender stigma and health: A critical review of stigma determinants, mechanisms, and interventions. Soc. Sci. Med..

[20] Crenshaw K. (1991). Mapping the margins: Intersectionality, identity politics, and violence against women of color. Stanf. Law Rev..

[21] Bowleg L. (2012). The problem with the phrase women and minorities: Intersectionality-an important theoretical framework for public health. Am. J. Public Health.

[22] Dang M., Scheim A.I., Teti M., Quinn K.G., Zarwell M., Petroll A.E., Horvath K.J., John S.A. (2022). Barriers and Facilitators to HIV Pre-Exposure Prophylaxis Uptake, Adherence, and Persistence Among Transgender Populations in the United States: A Systematic Review. AIDS Patient Care STDS.

[23] Teng F., Sha Y., Fletcher L.M., Welsch M., Burns P., Tang W. (2023). Barriers to uptake of PrEP across the continuum among transgender women: A global scoping review. Int. J. STD AIDS.

[24] Abreu R.L., Gonzalez K.A., Mosley D.V., Pulice-Farrow L., Adam A., Duberli F. (2022). “They feel empowered to discriminate against las chicas”: Latina transgender women’s experiences navigating the healthcare system. Int. J. Transgend. Health.

[25] D’Avanzo P.A., Bass S.B., Brajuha J., Gutierrez-Mock L., Ventriglia N., Wellington C., Sevelius J. (2019). Medical Mistrust and PrEP Perceptions Among Transgender Women: A Cluster Analysis. Behav. Med..

[26] Maiorana A., Sevelius J., Keatley J., Rebchook G. (2021). “She is Like a Sister to Me.” Gender-Affirming Services and Relationships are Key to the Implementation of HIV Care Engagement Interventions with Transgender Women of Color. AIDS Behav..

[27] Zamudio-Haas S., Koester K., Venegas L., Salinas A., Herrera C., Gutierrez-Mock L., Welborn L., Deutsch M.B., Sevelius J. (2023). “Entre Nosotras:” a qualitative study of a peer-led PrEP project for transgender latinas. BMC Health Serv. Res..

[28] Sevelius J.M., Keatley J., Calma N., Arnold E. (2016). ‘I am not a man’: Trans-specific barriers and facilitators to PrEP acceptability among transgender women. Glob. Public Health.

[29] Ogunbajo A., Storholm E.D., Ober A.J., Bogart L.M., Reback C.J., Flynn R., Lyman P., Morris S. (2021). Multilevel Barriers to HIV PrEP Uptake and Adherence Among Black and Hispanic/Latinx Transgender Women in Southern California. AIDS Behav..

[30] Aguayo-Romero R.A. (2021). (Re)centering Black Feminism Into Intersectionality Research. Am. J. Public Health.

[31] Wirtz A.L., Poteat T., Radix A., Althoff K.N., Cannon C.M., Wawrzyniak A.J., Cooney E., Mayer K.H., Beyrer C., Rodriguez A.E. (2019). American Cohort to Study HIV Acquisition Among Transgender Women in High-Risk Areas (The LITE Study): Protocol for a Multisite Prospective Cohort Study in the Eastern and Southern United States. JMIR Res. Protoc..

[32] Wirtz A.L., Cooney E.E., Stevenson M., Radix A., Poteat T., Wawrzyniak A.J., Cannon C.M., Schneider J.S., Haw J.S., Case J. (2021). Digital Epidemiologic Research on Multilevel Risks for HIV Acquisition and Other Health Outcomes Among Transgender Women in Eastern and Southern United States: Protocol for an Online Cohort. JMIR Res. Protoc..

[33] Creswell J.W., Vicki L., Plano Clark V.L. (2017). Designing and Conducting Mixed Methods Research.

[34] Hennink M., Kaiser B.N. (2022). Sample sizes for saturation in qualitative research: A systematic review of empirical tests. Soc. Sci. Med..

[35] Crabtree B.F., Miller W.L. (1999). Doing Qualitative Research.

[36] Benson C. (2020). Poverty: 2018 and 2019 American Community Survey Briefs. Tracking the rise of anti-trans bills in the U.S. https://www.census.gov/content/dam/Census/library/publications/2020/acs/acsbr20-04.pdf.

[37] Trans Legislation Tracker Tracking the Rise of Anti-Trans Bills in the U.S. https://translegislation.com/learn.

[38] The LGBTQ+ Bar (2025). Trump Anti-LGBTQ+ Executive Order Litigation Tracker. https://lgbtqbar.org/programs/trump-executive-order-tracker/.

[39] Page K.R., Polk S. (2017). Chilling Effect? Post-Election Health Care Use by Undocumented and Mixed-Status Families. N. Engl. J. Med..

[40] Perreira K.M., Pedroza J.M. (2019). Policies of Exclusion: Implications for the Health of Immigrants and Their Children. Annu. Rev. Public Health.

[41] Immigration Policy Tracking Project (2025). Tracking Trump Administration Immigration Policies. https://immpolicytracking.org/.

[42] Torres T.S., Nascimento A.R., Coelho L.E., Konda K.A., Vega-Ramirez E.H., Elorreaga O.A., Diaz-Sosa D., Hoagland B., Guanira J.V., Pimenta C. (2023). Preferences for PrEP modalities among gay, bisexual, and other men who have sex with men from Brazil, Mexico, and Peru: A cross-sectional study. Ther. Adv. Infect. Dis..

[43] Hernandez C.J., Santos G.M., Wilson E.C. (2020). Association of Documentation of Legal Residency Status with Nonprescribed Hormone Use Among Hispanic/Latina Trans Women in San Francisco. Health Equity.

[44] Sergi F.D., Wilson E.C. (2021). Filler Use Among Trans Women: Correlates of Feminizing Subcutaneous Injections and Their Health Consequences. Transgend. Health.

[45] Morris E., Teplinskaya A., Olansky E., Rinderle J.K., Chapin-Bardales J., National HIV Behavioral Surveillance Among Transgender Women Study Group (2024). Characteristics Associated with Pre-Exposure Prophylaxis Discussion and Use Among Transgender Women Without HIV Infection—National HIV Behavioral Surveillance Among Transgender Women, Seven Urban Areas, United States, 2019–2020. MMWR Suppl..

[46] Rivera A.V., Lopez J.M., Braunstein S.L. (2023). Exploring the Association Between Gender Affirmation and PrEP use Among Transgender Women in New York City. AIDS Behav..

[47] Cooney E.E., Reisner S.L., Poteat T., Althoff K.N., Radix A., Stevenson M., Wawrzyniak A.J., Cannon C., Schneider J.S., Mayer K. (2024). Interest in Long-acting Injectable PrEP Among Transgender Women in Eastern and Southern United States. JAIDS.

[48] Rhodes S.D., Alonzo J., Mann-Jackson L., Aviles L.R., Tanner A.E., Galindo C.A., Bessler P.A., Courtenay-Quirk C., Garcia M., Sucaldito A.D. (2024). Preexposure Prophylaxis Uptake Among Spanish-Speaking Transgender Women: A Randomized Controlled Trial in North and South Carolina, 2019–2022. Am. J. Public Health.

