# Supplementary material for: “When Somebody Comes into This Country and You Are Trans on Top of That Is Like You Got… Two Strikes on You”: Intersectional Barriers to PrEP Use Among Latina Transgender Women in the Eastern and Southern United States
Source: Int J Environ Res Public Health. 2025 Apr 22;22(5):659. doi: 10.3390/ijerph22050659 (PMC12111083; doi:10.3390/ijerph22050659)
Supplement: Supplementary file 1 [file ijerph-22-00659-s001.zip › ijerph-3514627-supplementary.pdf]

Supplementary Material File S1- The LITE American Cohort Study: Qualitative Interviews to explore the impact of individual, interpersonal, and structural factors on the participation of Latina transgender women (LTW)

Field Guide—Semi-Structured Interview Guide

Part 1: Self-Identity and Context: Community, Issues Facing LTW Living in the US, Health, and HIV Prevention and Treatment (20 min)

- Thinking about your identity and who you are, how do you define yourself?
- What terms do you use to capture your identity? (probes: Gender identity terms? Ethnoracial identity terms? Do you identify in the same way (gender identity/race) in your country of origin?
- To what extent is the term Latina transgender woman relevant for you or applicable to you?
- What is your level and type of involvement with LTW communities? To what extent do you feel connected with other LTW? (probes: Latinx transgender community? General Latinx community?)
- What challenges do you see LTW living in the U.S. face? (probes: language, employment, immigration, stigma)
- How might these challenges impact the health and wellbeing of LTW?
    - Probe: Access to healthcare?
- How might these challenges impact HIV prevention and HIV treatment specifically? (probes: HIV testing, PrEP use/adherence/persistence, PEP use, ART medication uptake and adherence, condom use).
    - Elicit a Concrete Example: If you feel comfortable, please share with me an experience from your own life where you had a challenge with HIV prevention or treatment. Describe the situation, challenge, and impact. If you cannot think of an experience in your own life, or prefer not to share, please consider sharing an experience you have heard from a LTW you know (a friend, peer) or from the LTW community.

Part 2: HIV Research Engagement (50 min)

A. General Perceptions of Research (10 min)

Next, I will ask about HIV research. When I say "HIV research" I am referring to: The systematic gathering of knowledge about HIV, including how to prevent it (for people who are HIV-negative) and how to treat it (for people living with HIV). HIV research can be pre-clinical or clinical (e.g., vaccines), and social and behavioral (e.g., social factors and behaviors that place people at-risk for HIV or protect them). HIV research includes studies that are qualitative (e.g., like this study involving interviews to gather voices of people to understand their experiences) and quantitative (e.g., surveys, numbers to characterize the HIV epidemic).

- What do you think of when you hear the term "HIV research"?
- What might the LTW community living in the U.S. think of when they hear the term "HIV research"?
- What are some of the risks of participating in HIV research for LTW?

- What are some of the benefits of participating in HIV research for LTW?
- What are some ways HIV researchers can ensure that their research is relevant for LTW?
- Thinking about all of the needs of the LTW community, where does HIV research fall in terms of priority and why?

B. Research Participation (10 min)

- Have you participated in other research studies <u>other than the LITE study</u>? (If No, jump to Question B1)
- Tell me a little about the research study (or studies) you have been involved in. *(Repeat set of questions below for each study mentioned)*
  - Was the study HIV research specifically?
  - How recently were you involved in the study?
  - What was being asked of you as a research participant?
  - What was your experience like participating in the research study?
    - Probe: Did you receive satisfactory information about the research and researchers? Did you trust the researcher(s)?
    - Probe: Did you trust that HIV researchers will keep your information confidential?
  - What did you like and dislike about participating in the study?
    - Probe: Compensation? Being treated ethically and respectfully? If participant mentions experiencing stigma or discrimination in research ask her what she attributes it to (e.g., her gender, race/ethnicity, class, nationality, or other).
  - Were the questions being asked specifically about Latina Transgender women?
  - In what ways was the study reflective of your experiences as a Latina transgender woman? Please, elaborate.
    - Probe: Content of questionnaires (or interviews)? What questions were missing? What questions could be improved? Quality of Spanish language translation?
- B1. What are some things that might encourage you to participate in HIV research studies now or in the future?
  - Probes: Incentives [what amount?] [Cash, physical gift card, virtual gift card, debit card, check] Transportation vouchers? A research team that includes LTW research team members? A research team led by LTW?
- What are some things that might discourage you from participating in HIV research studies now or in the future?

  Probes: Time commitment? Distrust of research in general? Composition of research team? Insufficient information about the study? Confidentiality concerns? Excessive focus on HIV? Competing priorities?
- Studies sometimes ask participants whether they would like to be contacted about future research or other studies. Do you feel comfortable being contacted about future research opportunities? Why or why not?

- ***Probes for LTW not born in the U.S. (not applicable to Puerto Rican Participants)***

Just a quick reminder that you can skip any questions you do not want to answer and also that as researchers, we will maintain all the personal information that you share secure and confidential and it cannot be used legally against you.

  - How does your immigration status impact your participation in research?
    - Probe: the ways you answer survey questions? (e.g., skipping questions, you do not always feel safe/protected to provide all the information that is asked of you, circumstances preventing disclosure of some information)
    - Probe if participant does not share immigration status: If you feel comfortable sharing, what is your immigration status?
    - Probe for undocumented participants: What are the risks you face when you participate in HIV research as an undocumented person?
      - Probe: Fear of deportation? Due to fear of deportation, how often have you avoided participating in HIV research?
  - What are some things that HIV researchers can do to decrease your fears when disclosing your immigration status?

- What has been your involvement in HIV research studies in roles other than as a research participant?
  - Probe: What roles (e.g., Community Advisory Board)?
  - How was your experience?
- What are some ways that you might want to be engaged in HIV research, other than being a participant? (Probe: design, participant recruitment, data collection, analysis, dissemination)?
- What do you recommend researchers do to make sure LTW participate in HIV research?
    - Probe: What should participants expect in return for participation in research?

C. Barriers and facilitators to research participation (10 min)

- What are some of the barriers (things that get in the way) of LTW participating in HIV research?
  - Probe Individual: Individual level barriers particularly for those born abroad can include language spoken, nationality, immigration/documentation status, years living in the U.S., reasons for migration, U.S. acculturation, fear of deportation, etc.
  - Probe Interpersonal: Interpersonal level barriers include stigma, experiences of discrimination and/or mistrust with researchers/health care professionals, etc.
- Probe Structural: Structural/systemic level barriers include lack of insurance, immigration laws, health care regulations, housing, employment, food insecurity, transportation access, etc. What are some of the things that can encourage or support (facilitators and motivators) LTW to participate in HIV research?
  - Probe Individual: Individual level facilitators can include language options, being U.S. born, etc.
  - Probe Interpersonal: Interpersonal facilitators include community networks, community social support, positive experiences with gender affirming researchers/health care professionals, etc.

    - Probe Structural: Structural/systemic level facilitators include access to care, access to CBOs, etc. (transphobia, racism, classism, xenophobia)
- What are some things that HIV researchers should consider when developing new studies for LTW?
    - Probes: Social, cultural, and historical considerations
    - Probe for cisgenderism, racism, xenophobia, classism, etc. in research
- What are some historical/political events or legal actions in the U.S. and in your country of birth (if born outside of the U.S.) that had an impact on LTW and how they view their participation in research?
    - Probe for cisgenderism, racism, xenophobia, classism, etc.
    - Probe for anti-trans bills in the U.S., xenophobia increasing since Trump's presidency

D. Participation modality preference (online vs. in person) (10 min)

- Preferences:
    - Do you prefer in-person or online participation in HIV research studies? Why?
    - How has the COVID-19 epidemic impacted your preference, if at all?
- Advantages and Disadvantages:
    - What are some of the advantages and disadvantages of in-person research participation for you? For LTW in general? (Probe for conflicting schedules with work commitment, option of RA help)
    - What are some of the advantages and disadvantages of online research participation for you? For LTW in general? (Probe for internet, computer, smartphone access and literacy)
    - What are some factors that might impact whether LTW participate in-person or online/remote? (Probe: technology access and comfort; transgender, ethnoracial, class stigma)
        - (Would RA assisted survey over the phone help if online survey is the only option and participant does not like it?)
- HIV/STI Testing Procedures:
    - For studies that include HIV/STI testing, do you prefer in-person/site-based testing, saliva test sent to labs or at home self-HIV/STI testing? Why?
    - How has the COVID-19 epidemic impacted your preference, if at all?
    - Do you think LTW in general prefer in-person/site-based testing, saliva test sent to labs, or at home self-HIV/STI testing? Why?
- Suggestions for future research:
    - What might HIV researchers need to consider when deciding between in-person and online/remote studies to ensure the participation of LTW? (For example, option to complete survey over the phone, transportation vouchers)

***E. Participation in LITE* (10 min)**

Select one question set for this section depending on participant engagement in LITE.

Group 1. Questions for LTW who only completed the baseline survey

- Why did you decide to complete the baseline survey?
- What were some of the reasons you participated in the baseline survey and not in the cohort?

    - Probe: Length of the survey? the required HIV/STI testing? Follow up visits? Long term commitment? Competing priorities?
- If those barriers could be addressed, would you be interested in continuing being part of the study? Why or why not?
- What could the research team have done to further engage you in the study?
- What other recommendations do you have for this research team and other HIV researchers interested in working with LTW in studies with multiple surveys over time?

Group 2. Questions for LTW who were lost to follow-ups

- Why did you decide to join the LITE cohort?
- What are the reasons why you are no longer participating in the LITE study?
    - Probe: Length of the survey? the required HIV/STI testing? Frequency of follow up visits? Long term commitment? Competing priorities?
- If those barriers could be addressed, would you be interested in continuing being part of the study? Why or why not?
- What could the research team have done to keep you in the study?
- What other recommendations do you have for this research team and other HIV researchers interested in working with LTW in studies with multiple surveys over time?

Group 3. Questions for LTW with intermittent follow-ups

- Why did you decide to join the LITE cohort?
- What are the reasons why you are not able to complete every follow-up survey/testing?
    - Probe: Length of the survey? the required HIV/STI testing? Frequency of follow up visits? Long term commitment? Competing priorities?
- If those barriers could be addressed, would you participate in every follow-up visit?
- What can the research team do to ensure your participation in every follow-up visit?
- What other recommendations do you have for this research team and other HIV researchers interested in working with LTW in studies with multiple surveys over time?

Group 4. Questions for LTW completing every time-point

- Why did you decide to join the LITE cohort?
- What motivates you to participate in every follow-up visit?
- What are the barriers that make it harder for you to participate in every follow-up visit?
- What else can the research team do to support you in continuing to participate in every follow-up visit?
- What other recommendations do you have for this research team and other HIV researchers interested in working with LTW in studies with multiple surveys over time?

F. Recruitment and Retention in Research (10 min)

<u>Recruitment:</u>

- Do you prefer to come across HIV research recruitment materials online or in-person? Why?
- Do you think recruitment online or in-person is more effective in reaching a larger number of LTW? Why?

- What do you think of the HIV research recruitment materials (flyers or announcements) that you come across that target LTW? (Probe: Have you seen any? Have they done a good job? How can they be improved?)
- As a Latina transgender woman, what do HIV researchers need to do for you to participate in a research study? (For example, mention availability in English and Spanish, compensation, etc.)
- What recommendations do you have for researchers that want to recruit LTW for HIV research studies?
- What are some of the in-person sites where you have found recruitment materials for HIV research? For example, nightclubs, community centers, health care centers, grocery stores.
- What are some of the online platforms where you have found recruitment materials for HIV research? For example, Facebook, Instagram, Twitter, WhatsApp.
- What additional recruitment online and in-person sites do you recommend HIV researchers use?

  <u>Retention</u>: Retention refers to staying a study over time and completing the end of the study.
- What are some of the barriers (things that get in the way) that LTW face staying in a study over time?
- What are some things that researchers could do to support LTW retention and staying in a study over time?

Part 3: PrEP Preference Qualitative Questions (5 min)

PrEP had only been approved and made available in pill form (Truvada or Descovy) for HIV prevention up until December 2021 when a long-acting injection (Apretude) was approved for use in transgender women as well. The pill and injection forms of PrEP are both highly effective at preventing HIV when taken as prescribed. The main difference is that the pill form requires you to take it daily, while the injection requires a shot every 2 months in the butt muscle by a clinical service provider in a clinic, hospital, and/or doctor's office.

· <u>Knowledge/Awareness</u>

· Have you heard of PrEP in injection form (before today)? Y/N.

· If yes: What have you heard? Where have you heard about injectable PrEP or come across information about it?

· If no: Would you be interested in learning more about it; why or why not?

o A. (If participant never used PrEP before- **PrEP group 1 or 2**) If you chose to start PrEP, do you prefer it in pill form or injection form? Why*? (If participant is interested in starting PrEP share PrEP locator link – preplocator.org)*

o B. (If currently using PrEP in pill form- **PrEP group 3**) Would you switch to the injection form of PrEP? Why?

o C. (If participant previously used oral PrEP – **PrEP group 4**) What were the barriers that made you stop using oral PrEP? (Probe for clinical visits, HIV testing, other screening for PrEP, taking daily pills felt excessive). Would using PrEP in injection form address those barriers?

o <u>Advantages and Disadvantages of PrEP Delivery Methods</u>:

- Pill: What are some of the advantages and disadvantages of PrEP in pill form for you? For TW in general? For LTW in general? (Probe for size of the pill, having to take it daily, convenience, privacy)
- Injection: What are some of the advantages and disadvantages of PrEP in injection form for you? For TW in general? For LTW in general? (Probe for fear of injection, familiarity with self-injecting hormones, Competence, convenience every 2 months, privacy)
- What are some factors that might impact whether you would choose PrEP in pill form or injection? TW? LTW? (Probe for injection preference to self-inject PrEP vs. provider initiated at health center)
- Scientists are also developing new PrEP methods that include implant, topical gel, IV Fluids (antibodies), in addition to pill and injection forms. All are as effective in preventing HIV. Which delivery method would you prefer and why?
- What would be the maximum amount that you would be willing to pay for PrEP per month?

Thank you for sharing your experiences and perspectives about participating in HIV research.

Part 4: Closing (5 min)

a. Is there anything else you would like to add?
b. Thank you for your generous participation in our study!
